# Effectiveness of a polyphenolic extract (*Lippia citriodora* and *Hibiscus sabdariffa*) on appetite regulation in overweight and obese grade I population: an 8-week randomized, double-blind, cross-over, placebo-controlled trial

**DOI:** 10.1007/s00394-021-02678-x

**Published:** 2021-09-30

**Authors:** Ana Serna, Javier Marhuenda, Raúl Arcusa, Silvia Pérez-Piñero, Maravillas Sánchez-Macarro, Ana María García-Muñoz, Desirée Victoria-Montesinos, Fernando Cánovas, F. Javier López-Román

**Affiliations:** 1 Faculty of Health Sciences, San Antonio Catholic University of Murcia (UCAM), 30107 Murcia, Spain; 2 Biomedical Research Institute of Murcia (IMIB-Arrixaca), Murcia, Spain

**Keywords:** *Hibiscus sabadarrifa*, *Lippia citriodora*, Appetite sensation, Polyphenols, Leptin

## Abstract

**Introduction:**

Polyphenols have shown capacity to improve appetite sensation, leading to enhanced control of body weight. However, despite being related with hunger-related hormones, metabolic and mechanism are not well known.

**Methods:**

The effectiveness of a nutraceutical composed of extract to *Lippia citriodora* and* Hibiscus sabdarrifa* (Lc-Hs) for controlling satiety and hunger was analyzed in a cross-over, placebo-controlled (Pla) clinical intervention. The study was divided in two 60-day periods separated by 30-day length wash-out period. At the end of each period, overweight and obese subjects (*n *= 33; age = 33.76 ± 12.23; BMI = 28.20 kg/m^2^ ± 2.47; fat mass 30.65 ± 8.39%; both sexes were proposed to eat an ad-libitum meal. Meanwhile, appetite sensation was determined by visual analog scales at different times. Moreover, blood extraction was performed to determine biochemical parameters (lipid and glucidic profile and safety parameters) and to evaluate hunger-related hormones (insulin, leptin, ghrelin, adiponectin, GLP-1 and peptide YY).

**Results:**

A decrease in appetite sensation was observed in Lc-Hs treatment, showing higher satiety quotient (Pla = 3.36 ± 2.33%mm/kcal; Lc-Hs = 5.53 ± 2.91%mm/kcal; *p *< 0.0001). Area under the curve was higher in Pla compared to Lc-Hs during the test, from baseline to minute 240 (240 (Pla 9136.65 ± 2261.46% *x* min^−1^; Lc-Hs 8279.73 ± 2745.71% *x* min^−1^; *p *< 0.014). Energy consumption was lower for subjects treated with Lc-Hs (774.44 ± 247.77 kcal) compared to those treated with Pla (849.52 ± 246.54 kcal) (*p *< 0.004). Leptin values varied from baseline (Pla 12.36 ± 1.98 ng/mL; Lc-Hs 13.13 ± 1.99 ng/mL) to the end of the study (Pla 12.60 ± 2.02 ng/mL; Lc-Hs 12.06 ± 2.05 ng/mL; *p *< 0.047). GLP-1 values varied (*p *< 0.001) in Lc-Hs treatment from baseline (4.34 ± 0.49 ng/mL) to the end of the study (3.23 ± 0.52 ng/mL).

**Conclusion:**

The supplementation with the Lc-Hs extract decreases appetite sensation in overweight and obese population, reducing calorie intake after an ad-libitum meal. Due to variation on hunger-related hormones and the relationship between satiety feeling, it would be interesting to develop future research focused on the variation of the hormones themselves.

## Introduction

According to the World Health Organization, overweight and obesity are known as abnormal or excessive accumulation of body fat that is harmful to health, and unfortunately has become a worldwide epidemic in the twenty-first century, especially in the last decades [1–3]. Excess of body fat, is widely associated with non-communicable diseases including; hypertension, type II diabetes, cardiovascular diseases, hyperlipidemic, non-alcoholic fatty liver and various types of cancer [4–7]. An increase in fat mass could be summarized in a reductionist way as an imbalance between caloric intake and energy expenditure. However, it may also due to many factors such as genetics or psychological disorders [7]. Natural products are an alternative to pharmacological drugs and can represent a fate alternative with minimal or no side effects [8, 9].

Appetite plays an important role in food consumption, influencing hunger and therefore caloric intake [10]. It is defined as desire to consume food, which disappears once food intake takes place and it is followed by satiety feeling [11]. Such sensations are triggered in the hypothalamus, which regulates the centers of hunger and satiety [12] which have neurotransmitters and hormones receptors that modulate eating behavior. These substances include those that stimulate appetite (orexigenics) such as ghrelin, and those that inhibit it (anorexigenic) such as insulin, leptin, adiponectine, peptide YY and GLP-1 [13].

The interest in plant-derived polyphenols are due to their antiviral, antitumoral, antiatherogenic, anti-inflammatory, antihypertensive, antilipogenic and antioxidant capacities [14–16]. Scientific literature shown that certain plant-derived extracts such a *Hibiscus sabdariffa*, *Lippia citriodora* extracts can modulate different metabolic pathways and activating the AMPK pathway which favor lipolysis and, therefore, fat loss [17–19], an enzyme complex considered as a cellular energy detector that helps energy balance of the cell and the total consumption of kilocalories.

AMPK has been shown to cause the inactivation of acetyl-CoA carboxylase, 3-hydroxy-3-methylglutaryl (HMG)-CoA reductase, the key regulatory enzymes of cholesterol and fatty acid synthesis [20, 21].

AMPK is expressed throughout the brain, including several areas that control food intake and neuroendocrine function, such as the hypothalamus and the rhombencephalon [22, 23]. Hypothalamic AMPK is influenced by energy intake and availability, as evidenced by the fact that fasting increases and eating decreases its activity in the hypothalamus, as well as being affected by various orexigenic and anorexigenic signals from this site. Leptin has a specific effect on AMPK: in skeletal muscle, it stimulates its activity while, in the hypothalamus, it has the opposite effect, decreasing the activity of hypothalamic AMPK. These apparently paradoxical tissue-specific effects of leptin contribute to the overall positive effect of this hormone on energy homeostasis, which leads to increased oxidation of fatty acids in peripheral tissue [24, 25] as well as reduced appetite in the hypothalamus with the consequent reduction in body weight.

These polyphenolic extracts contained in the product could help reduce fat mass by modulating the activity of AMPK and, thereby, normalize the levels of leptin that are usually elevated in people who are overweight or obese, due to a resistance to this hormone and to decrease in circulating soluble leptin receptors (LSC) [26].

We hypothesized that chronic consumption of this polyphenolic extract composed of *Lippia citriodora* and *Hibiscus sabdariffa* (Lc-Hs) could have effects on appetite regulation in overweight and obese people.

## Materials and methods

### Trial design

**Fig. 1:**
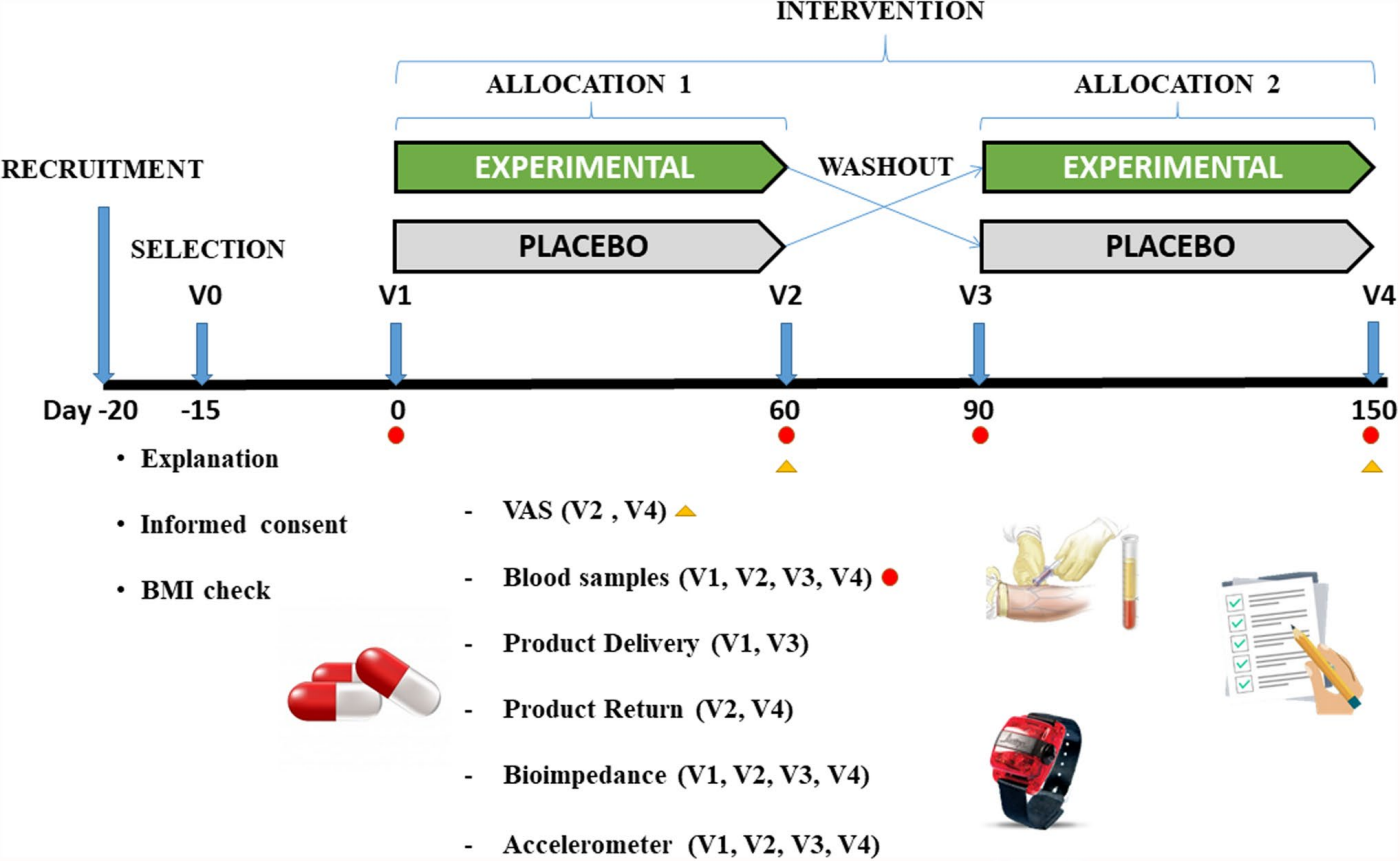
Graphic representation of the study design and some variables. Letter V means visit

The study consisted of a randomized controlled trial, double-blind, cross-over clinical trial with two branches depending on the product consumed experimental (Lc-Hs) or placebo (Pla) and single center (Fig. 1).

The present study had a length of 150 days divided into two allocations of 60 days separated by a wash-out period of 30 days. In the first phase subjects consumed the Lc-Hs or Pla and after the wash-out period the treatments were crossed. Each subject was assigned a code generated by a software generator (Epidat v4.1 Epidat, Spain) and was randomized in a 1:1 ratio to either of the two treatments (Lc-HS or placebo). Both the researchers and the subjects did not know which treatment subjects received.

### Participants

**Fig. 2:**
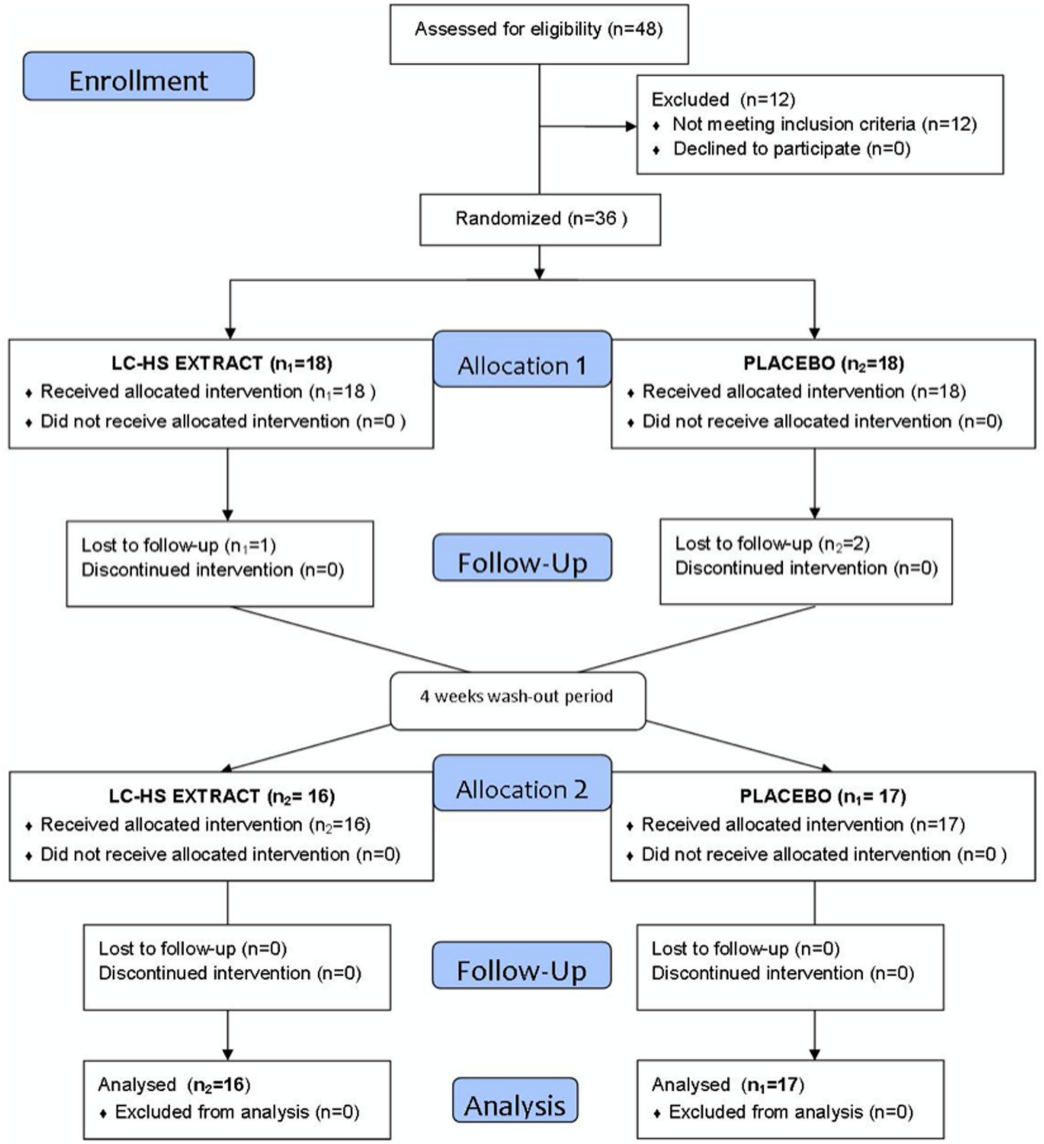
Flowchart

A total of 36 healthy subjects of both sexes participated in the study, of which 33 completed the intervention and were included in the final analysis as depicted in Fig. 2 (2 of the participants were unable to attend the appointment at the appropriate time and 1 did not want to continue with the study for personal reasons). Participants were recruited disseminating information on the study through talks in women’s centers, senior centers, neighborhood associations, and mass media. All subjects selected for possible inclusion were cited for a first face-to-face selection visit (15 days prior to the start of the study), where they were fully and in-depth informed of all pertinent aspects of the study (nature and objectives), as well as the possible risks involved, including written information and opinion favorable of the ethics committee. If the subject decided to participate in the study, their informed consent was obtained, freely granted and in writing before the realization of any study-related activity.

Subjects had to meet all inclusion criteria (age between 18 and 65 years old, both sexes, body mass index (BMI) between 25 and 34.9 kg/m^2^, weight maintained in the last three months, do not modify nicotinic habits and absence of disease) and any of the exclusion criteria (chronic diseases, illness, thyroid dysfunction, allergies to any components of the study product, carrying out or intend to carry out any type of diet during the study and participants that are in pregnancy state). A total of 33 overweight and obese subjects; 17 males and 16 females were part of the study, with a mean age of 33.76 ± 12.23 years, a weight of 82.54 ± 11.38 kg, a BMI 28.20 ± 2.47 and 30.65 ± 8.39% of fat mass at baseline. Once the participant signed the informed consent and was included in the study (after analyzing the aforementioned criteria), the researchers indicated the protocol to follow to the participants and were instructed to take two capsules a day.

The study protocol was approved by the Institutional Review Committee of the Universidad Católica San Antonio (Murcia, Spain; CE011815). Furthermore, the study was conducted in accordance with the Declaration of Helsinki [Randomized Trial Registration Number (Clinicaltrials): NCT04105192].

### Test products

Lc-Hs (MetabolAid^®^, Monteleloeder S.L., Alicante, Spain) and Pla presented similar pharmaceutical form. Lc-Hs was composed by a mixture of extracts from *Lippia citriodora* and *Hibiscus sabdariffa*, while Pla contained crystalline microcellulose. The administration was oral consisting on 2 capsules/day, each capsule containing 250 mg of either Lc-Hs (the two capsules correspond to the daily dose of 325 mg Lc and 125 mg Hs) or Placebo, before breakfast during a period of 60 days. At the end of each allocation, subjects were asked to return the empty blisters to check compliance of supplementation protocol. Same procedure was repeated after the cross-over.

The composition of the formula and HPLC data has been published in previous reports [9, 17]. Briefly, *Lemon verbena* and *Hibiscus extracts*, standardized in verbascoside and anthocyanins, respectively, are mixed in a 65–35% ratio.

**Fig. 3:**
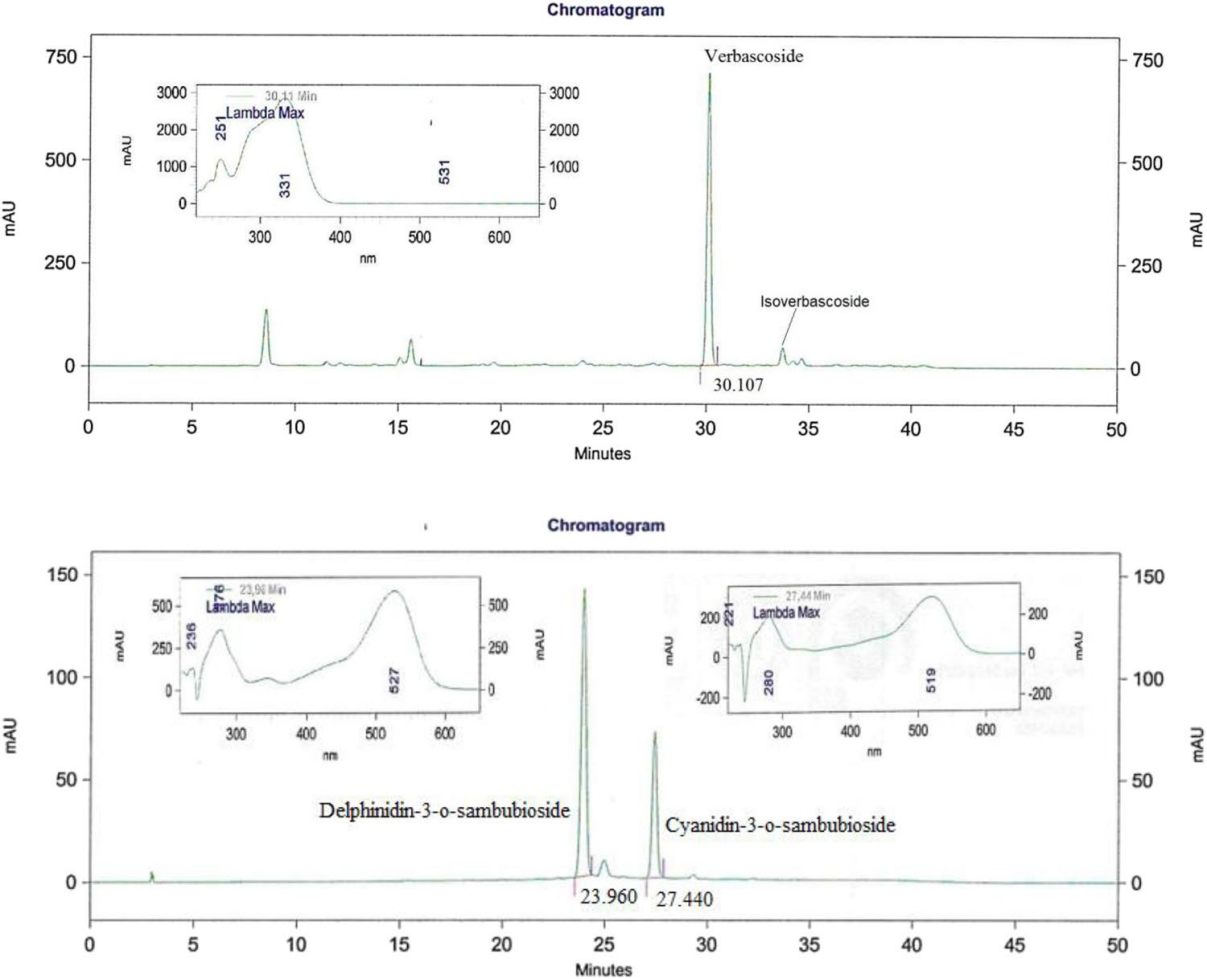
Representative HPLC chromatograms of the LC-HS combination at 320 nm for phenylpropanoids (upper panel) and 520 nm for anthocyanins (lower panel). Four compounds including delphinidin-3-O-sambubioside, cyanidin-3-O-sambubioside, verbascoside and isoverbascoside were determined by HPLC

Four major phenolic compounds are identified, i.e. two anthocyanins, delphinidin-3-O-sambubioside and cyanidin-3-O-sambubioside (from the *Hibiscus* extract) and two phenylpropanoids, verbascoside, and isoverbascoside (from the *Lemon verbena* extract). Total anthocyanins represent 3.5% of the total dry weight with delphinidin-3-O-sambubioside constituting 2.27% (65% of total anthocyanidins) and cyanidin-3-O-sambubioside comprising 1.23% (35% of total anthocyanidins). Regarding phenylpropanoids, 16% w/w, verbascoside represents the major compound and constitutes 15% (93.75% of total phenylpropanoids) and isoverbascoside represents 1% (6.65% of total phenylpropanoids). Figure 3 below taken from Herranz-Lopez et al. [17].

Comparative analysis was performed in a previously published report, by analyzing the effect of *Lemon verbena* extract, *Hibiscus* extract, its combination (the studied ingredient), and *Garcinia cambogia*, in high-fat diet-fed mice [9]. *Garcinia cambogia* is a widely extended plant extract consumed for its weight loss properties. The published study revealed that the combination of *Lemon verbena* and *Hibiscus* extracts, in the proportions found in the present ingredient, provide numerous benefits over *Garcinia cambogia* and the extracts in isolation. This includes lower body weight gain in presence of a high-fat diet, higher adiponectin expression, and lower leptin expression; all using a lower concentration of ingredient (100 mg/kg vs 245 mg/kg with *Garcinia cambogia*).

Other authors have also reported the effect of *Hibiscus* and *Lemon verbena* on some of the analyzed hormones, such as *Hibiscus* on leptin expression [27], or *Lemon verbena* activating AMPK [28], which is known to have an important role in controlling appetite in the hypothalamus [29]. Furthermore, high polyphenolic intake has been associated with weight loss, particularly anthocyanins and flavonols [30].

### Study settings

Subjects came to laboratory several times. Study design is depicted in Fig. 1. In each phase, subjects visited to the laboratory two times. At baseline of each phase (Visit 1, Visit 3), blood samples (3 mL) were obtained from the antecubital veins of subjects to determinate hormonal and biochemistry (lipid and glucidic profile and safety parameters) analysis. After blood extraction, each subject received the product to consume (Lc-Hs or Pla) and an accelerometer was placed on the non-dominant wrist (ActiGraph wGT3-BT. ActiGraph, Pensacola, FL, USA) to evaluate their physical activity. The monitor was configured to save data for 3 consecutive days. The participant wore it all the time except during water-based activities. The intensity of physical activity (sedentary, light, moderate and vigorous) was determined using Freedson's algorithm and categories [31]. Physical activity was expressed in METs/day. The software used in the accelerometer data analysis was ActiLife 6 (ActiGraph, Pensacola, FL; USA).

Once the product was delivered and the subjects were instructed, all participants were measured for standing height using ISAK protocols [32]. Subsequently, a foot-to-foot bioimpedance (Tanita BC-420M. Tanita Corporation, Arlington Heights, IL, USA) was performed to evaluate possible changes in body composition (weight, BMI, fat mass, and muscle mass). Subjects had to arrive after a 12 h fasting period, only allowing water intake until the previous 3 h. In case of smokers, the last cigarette should have been smoked at least 1 h before extraction. Moderate–high-intensity exercise could not be performed in the previous 24 h. The standardized conditions with respect to body position, previous exercise, dietary intake and body hydration have been respected [33]. At the end of each phase (Visit2, Visit4), blood collection (3 mL), evaluation of physical activity and body composition analysis were performed again.

To determine the efficacy of Lc-Hs, ad-libitum appetite test and visual analog scale (VAS) were performed at the end of the two phases (Visit 2 and Visit 4). During these visits, subjects came to the laboratory on fasted and took the product 30 min before eating a standardized breakfast. Before the participants attended visits Visit 2 and Visit 4, considering their breakfast time, the time from which they could not eat food was calculated. When they arrived at the established time, they were asked the time of the last meal and it was verified that they complied with the established recommendations. After subjects ate, blood samples (3 mL) were taken and VAS were filled by subjects at baseline, which would continue until 270 min (after consumption of the breakfast) at different times (0, 15, 30, 60, 90, 120, 180, 240, 270). Participants had to eat all breakfast and had a maximum time of 15 min. At minute 240, an ad-libitum buffet was served to quantify an ad-libitum intake. Nutritional value of the food served was determined using Dietsource v3.0 software (Novartis Medical Nutrition S.A., 1997–2003 which uses a table of composition of Spanish foods [34]. The breakfast was composed by bread (50 g), ham (30 g), cheese (25 g), pineapple juice (200 mL) and non-flavored yogurt (125 g), presenting a nutritional value of 417 kcal, 47 g of carbohydrates, 2 g of fiber, 21 g of protein and 15 g of lipids.

Later, in the ad-libitum meal, the subjects chose the type of food (fresh potato omelette with onion, meatballs with tomato, peas with ham, bolognese macaroni, vegetable stew and white toast), and this was weighed before and after the buffet lunch intake. They had a maximum time of 30 min for the ad-libitum buffet. Therefore, energy and macronutrients intake were determined for every volunteer from the quantitative measurement of food ingested.

### Main variable: satiety assessment

Visual analog scales (VAS) were used to evaluate Appetite Score (AS) after the intake of every product under investigation. Subjects reported their state of hunger, satiety and fullness on a 100 mm VAS by placing a vertical line on a scale. The VAS ranged from “not at all” and “extremely” and was divided in four questions to assess hunger (how hungry do you feel?), desire to eat (how strong is your desire to eat?) fullness (how full do you feel?) and prospective food consumption (PFC) (how much food do you think you can (or you want) eat?). AS was calculated as the average of the four individual scores: $\left( {{\text{Satiety}}\; + \;{\text{fullness}}\; + \;{\text{PFC}}\; + \;\left( {100 - {\text{hunger}}} \right)} \right)/4$. That formula and the interpretation of the results were adapted from previous studies [35], concluding that lower AS corresponds to greater fullness.

**Fig. 4:**
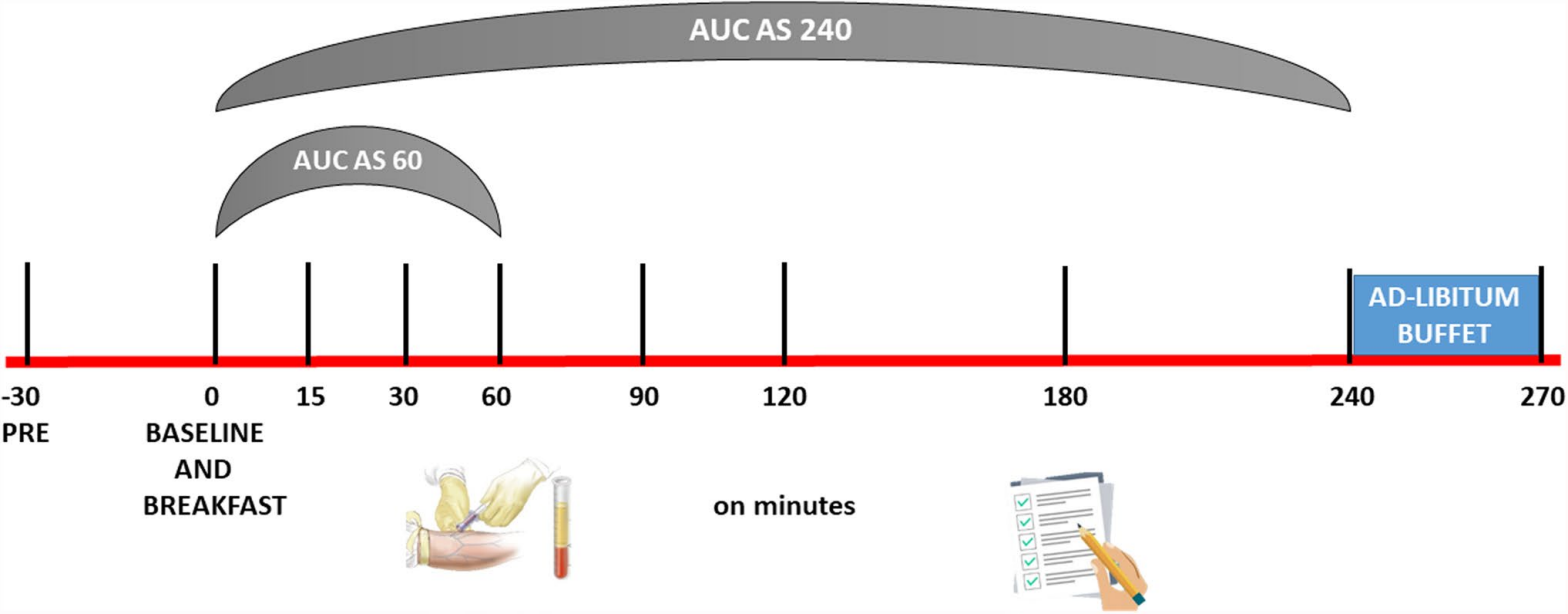
Instant on minutes (from baseline to min 270) where blood samples and VAS were performed during the study

VAS for each of the evaluated components of appetite, were completed by subjects in the following times: before and immediately after the intake of product under investigation, and at 10″, 20″, 30″, 40″, 50″, 60″, 120″, 180″, 240″ (immediately before ad-libitum meal) and 270″ post-intake of foods under investigation (immediately after consumption of ad-libitum meal) (Fig. 4) [36, 37].

In the measurement of AS, the area under the curve was calculated by summarizing the mean scores of pairs of adjacent time points and then calculating a weighted mean (weighted by the time difference of two time points). AS was calculated at every measurement interval, and the change in AS was calculated as the difference between AS baseline and AS of each subsequent measurement using the following formula: ${\text{Decrease}}\;{\text{of}}\;{\text{appetite}}\; = \;{\text{AS}}\;{\text{pre - intake}} - {\text{AS}}\;{\text{post - intake}}$; in which the average AS 60 min post-intake was represented by the area under curve (AUC) at AS (time 0, 10, 20, 30, 40, 50 y 60). AUC was measured by the area under the curve of time course of AS from minute 0 to 60 post-intake of product.

Satiety evaluation, using VAS was carried out for each product in every volunteer who attended the present research. The later term effect on satiety of a food was determined from VAS, and the energy composition of products contained in the ad-libitum food consumption test, using the formula of satiety quotient (SQ). The implementation of SQ is an additional aid to the determination of the satiating capacity of food, and provides information on the capacity of a food, a nutraceutical or a short-term appetite control drug [38].

SQ was calculated by the subtraction of the value AS before the intake of the product under study, minus the average of AS in the 60 min after intake of the product. This difference was divided by the energy values of the ingested ad-libitum food. By convention, the result of SQ is multiplied by 100 to obtain a more manageable range of values. Therefore, the resulting formula was: SQ (% mm/Kcal) = Decrease of appetite (defined as: (AS pre-intake − Average AS 60 min post-intake)/Energy of products under investigation (kcal) × 100), following the protocol used by Drapeau et al. [39, 40] only adapting the scale from 150 to 100 mm. As shown in the following meta-analysis, different types of scales can be used without affecting SQ [41]. Regarding the interpretation of SQ values, higher SQ, greater satiety.

### Biochemical analysis

Blood samples were collected in hemograme (tube w. K3 EDTA for 3 mL lavender cap 13 × 75 mm; Vacutest) and biochemistry (tube with gel and clot activate Vol 3.5 mL 13 × 75 mm; Vacutest) tubes and centrifuged (4500 rpm, 5 min, 4 °C) and then stored at − 80 °C and subsequently analyzed to monitor satiety hormones [insulin, leptin, adiponectin, ghrelin, peptide tyrosine-tyrosine (PYY) and Glucagon-like Peptide-1 (GLP-1)], glycemic profile [glycemia, insulinemia, glycated hemoglobin, peripheral insulin resistance (HOMA-R)] and lipid profile (total cholesterol, LDL cholesterol, HDL cholesterol, triglycerides). The methods used to analyze satiety hormones were: leptin [Human Adiponectin ELISA Kit; catalog no: RD195023100; sensitivity 26 ng/mL; lower limit of detection 0.1 µg/mL; inter-assay variation 5.6%; intra-assay variation 5.9%; BioVendor Research & Diagnostic Products (Brno, Czech Republic, Europe)], adiponectin [Human Leptin ELISA Kit; catalog no: RD191001100; sensitivity 0.2 ng/mL; lower limit of detection 1 ng/mL; inter-assay variation 6.7%; intra-assay variation 4.9%; BioVendor Research & Diagnostic Products (Brno, Czech Republic, Europe)], Ghrelin [Human GHRL (Ghrelin) ELISA Kit; catalog no: E-EL-H1919 96T; sensitivity 0.1 ng/mL; lower limit of detection 0.16 ng/mL; inter-assay variation 4.7%; intra assay variation 4.5%; Elabscience biotechnology Inc (Wuhan, Hubei, China)], peptide tyrosine-tyrosine [Human PYY (Peptide YY) ELISA Kit; catalog no: E-EL-H1237 96T; sensitivity 18.65 pg/mL; lower limit of detection 31.25 pg/mL; inter-assay variation: 5.1%; intra-assay variation 5.2%; Elabscience biotechnology Inc (Wuhan, Hubei, China)], Glucagon-Like Peptide-1 [Human GLP-1 (Glucagon Like Peptide-1) ELISA Kit; catalog no: E-EL-H0148 96T; sensitivity 0.19 ng/mL; lower limit of detection 0.31 ng/mL; inter-assay variation 5.5%; intra-assay variation 5.9%; Elabscience biotechnology Inc (Wuhan, Hubei, China)]. BA400 automatic clinical chemistry analyzer (BioSystems S.A, Costa Brava, Barcelona, Spain) was used to obtain the glycemic and lipidemic values.

### Statistical analysis

The analysis was performed triple blind. For quantitative variables, t-Student comparison was developed between both branches of the study, prior checking normality of the values (Shapiro–Wilk test; *n *< 50). To analyze the differences between treatments (Lc-Hs and Pla) and sexes, a variance analysis for repeated measures (rANOVA) were carried out with two intra-subject factors (time: basal and final or basal, 0, 15, 30, 60, 90, 120, 180, 240 and 270 and product: Lc-Hs). In this way, differences were established in each of the variables analyzed, considering these factors. Moreover, Bonferroni test was performed for post hoc analysis. The significance level used was 0.05, and statistical analysis was carried out with the SPSS 21.0 software and R-software.

## Results

As shown in Table 1, the basal sensation of appetite did not show significant differences between the two groups (*p* > 0.05). The AUC of appetite evolution during the first hour post-ingestion, as well as the mean appetite sensation during the first hour post-ingestion was significantly lower (*p* < 0.001) in the group consuming extract versus the control group. The decrease in appetite sensation and satiety quotient was significantly higher (*p* < 0 0.0001) for the group that consumed the extract versus the control group. Regarding the amount of energy consumed (kcal) in the ad-libitum intake, it was significantly higher (*p* < 0.004) in the control group than in the extract group and when evaluating the sensation of appetite after ad-libitum intake, no significant differences were observed between groups (*p* > 0.05).

**Table 1:** Variables measured in the study

	Control	Extract
Basal appetite sensation (%)	69.44 ± 14.76	73.09 ± 12.89
AUC of appetite evolution 0–60 min post-ingestion (% *x* min^−1^)^b^	2565.72 ± 792.33	2078.67 ± 898.99
Mean appetite sensation 0–60 min post-ingestion (%)^b^	42.76 ± 13.21	34.65 ± 14.98
Decrease in appetite sensation when consuming the product (%)^c^	26.68 ± 16.00	38.45 ± 16.96
Amount of energy consumed (Kcal)^a^	849.52 ± 246.54	774.44 ± 247.77
Appetite sensation immediately post meal ad-libitum (%)	16.92 ± 8.85	16.16 ± 8.85
SQ (% mm/Kcal)^c^	3.36 ± 2.33	5.53 ± 2.91

^a^Means significant statistical differences *p* < 0.004 between treatments

^b^Means significant statistical differences *p* < 0.001 between treatments

^c^Means significant statistical differences *p* < 0.0001 between treatments

### Satiety assessment

**Fig. 5:**
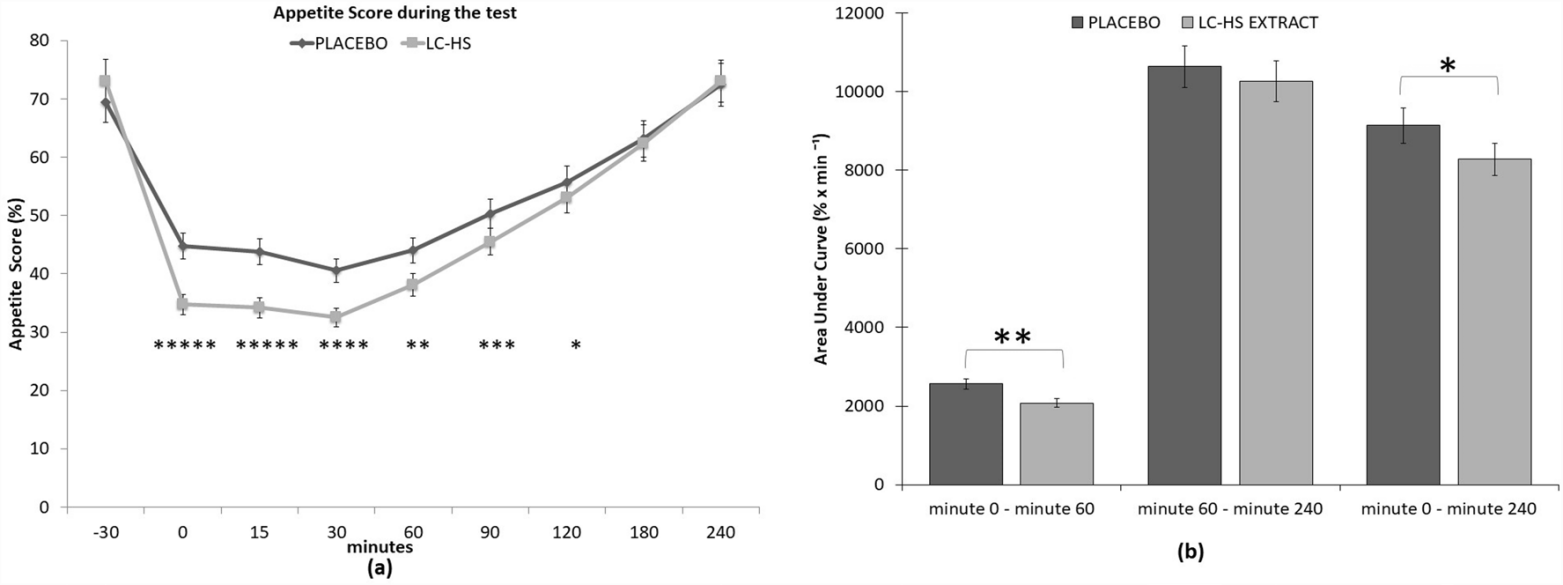
**a** Satiety quotient % and **b** Decline of AS average from baseline to minute 60. *Means significant statistical differences between treatments *p* < 0.0001

Due to the high predictive capacity, the main variable for the study of satiety was SQ. This quotient relates food intake to motivation to eat in the period after food intake, a relationship that cannot be determined by individual analysis of the amount of food consumed or ratings of motivation to eat. Interestingly, the treatment with the Lc-Hs extract resulted in enhanced SQ, leading to higher value compared with subjects from Pla treatment (*p* < 0.0001). Therefore, SQ reported from the Lc-Hs treatment was 5.53 ± 2.91%mm/kcal compared to the subjects from Pla treatment who reported minor value (3.36 ± 2.33%/kcal) (Fig. 5).

**Fig. 6:**
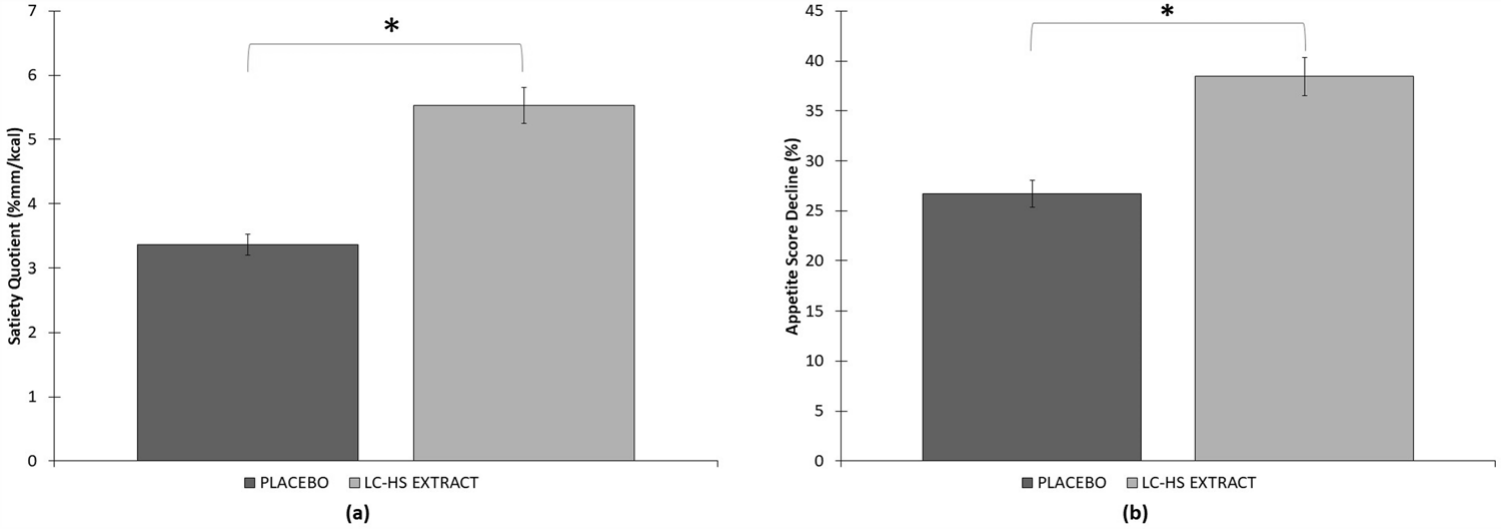
**a** Appetite Sensation in different moments of the study until min 240. *Means significant differences between treatments *p* < 0.02. **Means significant differences between treatments *p* < 0.005. ***Means significant differences between treatments *p* < 0.002. ****Means significant differences between treatments *p* < 0.001. *****Means significant differences between treatments *p* < 0.0001. **b** Area Under the Curve from placebo and extract. *Means significant statistical differences *p* < 0.05. **Means significant differences between treatments *p* < 0.001

The remaining parameters to determine satiety also showed interesting results, being improved after treatment with Lc-Hs. Both Lc-Hs and Pla treatments showed similar appetite sensation (AS) and satiety sensation parameters at baseline. However, after VAS accomplishment by subjects, results showed that subjects treated with Lc-Hs felt fuller compared to those treated with Pla. After a standardized breakfast intake at baseline (min − 30), VAS scales were given to the subjects. During the first monitoring 2 h, it was found significant difference at the different control times during the scales, comparing Lc-Hs and Pla treatments [min 0: Pla (44.78 ± 6.92%) compared to Lc-Hs (34.78 ± 8.82%) (*p* < 0.0001), min 15: Pla (43.77 ± 8.06%) compared to Lc-Hs (34.2 ± 8.75%) (*p* < 0.0001), min 30: Pla (40.58 ± 9.33%) compared to Lc-Hs (32.54 ± 8.90%) (*p *< 0.001), min 60: Pla (44.01 ± 7.93%) compared to Lc-Hs (38.13 ± 9.29%) compared to Lc-Hs (*p *< 0.005), min 90: Pla (50.31 ± 8.2%) compared to Lc-Hs (45.53 ± 8.97%) (*p* < 0.002), min 120: Pla (55.7 ± 7.42%) compared to Lc-Hs (53.09 ± 9.2%) compared to Lc-Hs (*p* < 0.018)]. The evolution of AS and satiety is depicted in Fig. 6.

According to the aforementioned data, a large decrease evaluating the average of AS during the first hour was observed in subjects treated with Lc-Hs compared to those treated with Pla (*p* < 0.0001). Specifically, AS decreased from 73.09 ± 12.89% to 34.65 ± 14.98% in Lc-Hs and from 69.44 ± 14.76% to 42.76 ± 13.21% in Pla. That variation is depicted Fig. 5.

AUC scores also showed the same correlation than the observed for satiety and AS. In this case, analyzing different periods of the test, increased AUC was observed in subjects treated with Pla compared to those treated with Lc-Hs. During the first hour (*p* < 0.0001), AUC was higher in Pla subjects (2565.72 ± 792.33% min^−1^), while Lc-Hs treatments showed minor AUC (2078.67 ± 898.99% min^−1^). However, the differences in AUC were decreasing as the minutes passed. Therefore, in the interval from min 60 to min 240 (*p* = 0.249) AUC of Pla (10,638.92 ± 2413.30% min^−1^) and Lc-Hs (102,565.65 ± 2940.48% min^−1^) treatments showed to be close. Finally, due to the marked difference between treatment treatments in the first 60 min, the overall AUC from baseline to min 240 (*p* < 0.014) was 9136.65 ± 2261.46% min^−1^ for Pla and 8279.73 ± 2745.71% min^−1^ for Lc-Hs.

It was observed that Lc-Hs treatment reported enhanced satiety and minor appetite than subjects treated with Pla, indicating better satiety response. Furthermore, despite the similar values observed of both treatment treatments observed in the interval from min 60 to min 240, the marked difference observed during the first hours lead to statistically significant differences along the following 4 h (Fig. 6).

**Fig. 7:**
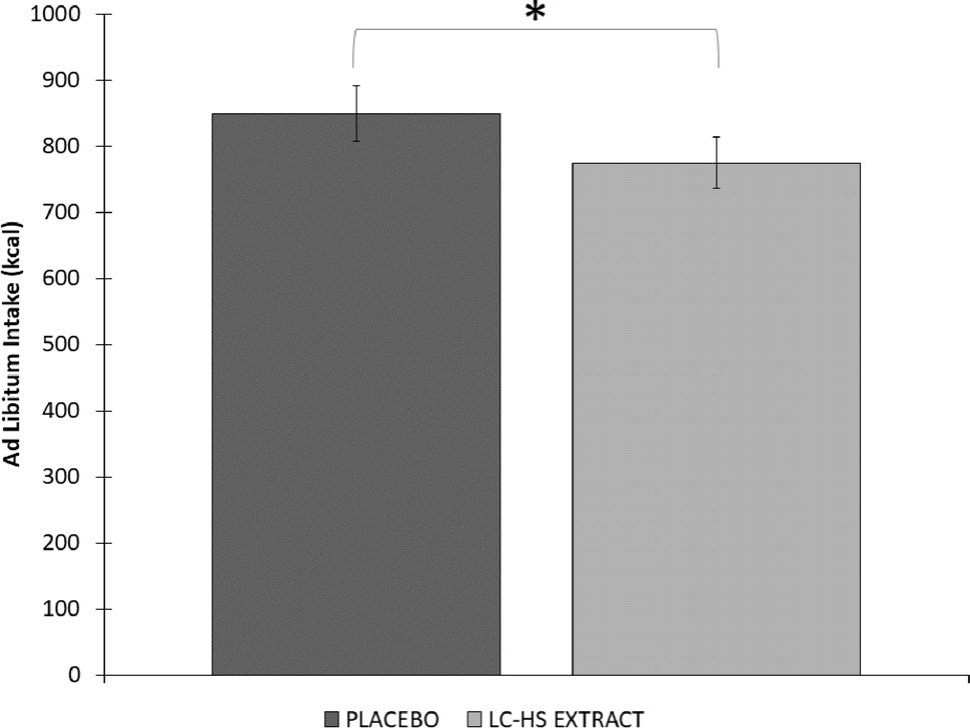
Quantity of kcal intake at minute 270 (Ad-libitum intake). *Means significant statistical differences between treatments *p* < 0.004

Finally, the caloric consumption during ad-libitum meal at the end of the 4 h, showed minor (*p* < 0.004) energy consumption for subjects treated with Lc-Hs (774.44 ± 247.77 kcal) compared to those treated with Pla (849.52 ± 246.54 kcal) (Fig. 7). These data together with SQ, reflect the relevance of AS and satiety as key factors in the number of calories consumed in a meal and the consequent feeling of satiety after that meal. Macronutrients content after ad-libitum intake was similar in both treatments, leading to non-significant differences between them. The intake of protein, carbohydrates and fats in Pla treatment was 32.4 ± 16.59, 141.75 ± 75.21 and 44.3 ± 18.65 g, respectively. In turn, the Lc-Hs treatment reported 34.95 ± 18.84 g of protein 140.95 ± 81.85 g of carbohydrates and 46.30 ± 20.84 g of fats. The differences observed in macronutrients may be attributed due to inclusion of dietary fiber (mainly cellulose) as carbohydrates.

Finally, the differences regarding sex reported interesting conclusions. Actually, there are differences by sex but they are not statistically evident. As the population between men and women rises, the number of volunteers in each group becomes too small and significant differences are lost.

Studying the SQ in both sexes, a significant rise in the satiety quotient is observed after consumption of Lc-Hs. However, it can be seen that in women this increase is greater, although when comparing the changes of this variable between men and women, no significant differences are seen (*p* = 0.107).

The mean AUC at 240 min shows that the decrease after the consumption of Lc-Hs is not significant in men (*p* = 0.58) and if it is significant in women (*p* = 0.015). But when comparing these differences, they are not significant (*p* = 0.152) given the small number of volunteers in each group. In summary, for leptin and GLP-1, the levels were higher in women than in men. The modifications observed when the experimental product is consumed in men is not significant for either of the two incretins, but in women it is. However, when comparing men with women, no differences were observed (*p* > 0.05). Moreover, Ghrelin did not show any difference between sexes (*p* > 0.05).

### Hormonal analysis

To carry out a hormonal analysis of satiety, both a long-term control during the study (before and after 60 days of product consumption) and a short-term control during the test were carried out.

Baseline values were similar in both treatments for all hormones (*p* > 0.05). Table 2 shows the values obtained for long-term hormonal analysis. Anorexigenic hormones as insulin, adiponectin and PYY did not showed statistically significant intra-treatment variation, neither in Lc-Hs or Pla treatments (*p* < 0.05). However, leptin showed significant differences between Lc-Hs and Pla treatments (*p* < 0.047), leading to minor leptin synthesis in Lc-Hs subjects (12.06 ± 2.05 ng/mL) than Pla treatment (12.60 ± 2.02 ng/mL). In case of GLP-1, significant differences were observed comparing both Lc-Hs and Pla treatments separately, leading to higher synthesis after the treatment of Lc-Hs treatment (*p* < 0.05). However, there was not found any difference in subjects treated with Pla. On the other hand, the orexigenic hormone ghrelin—commonly known as the hunger hormone [42]—did not suffer statistically significant variation in neither Lc-Hs nor Pla during the study. However, a downward trend was observed in the subjects from the Lc-Hs treatment. In addition, the determination of insulin followed similar trend than the glycemic curve, according to plasmatic glucose concentration.

**Table 2:** Evolution of hormonal analysis of subjects during the study

	Baseline	Final
Insulinemia (mU/L)		
Control	8.11 ± 0.70	8.62 ± 0.77
Extract	7.56 ± 0.47	7.77 ± 0.54
Leptin (ng/dL)^a^		
Control	12.36 ± 1.98	12.60 ± 2.02
Extract	13.13 ± 1.99	12.06 ± 2.05
Adiponectin (µg/mL)		
Control	8.86 ± 0.55	8.64 ± 0.59
Extract	8.58 ± 0.56	8.42 ± 0.55
Ghrelin (ng/mL)		
Control	4.06 ± 0.54	4.11 ± 0.49
Extract	4.08 ± 3.87	3.87 ± .056
PYY (pg/mL)		
Control	55.27 ± 8.93	52.79 ± 9.45
Extract	54.54 ± 9.90	53.89 ± 11.74
GLP-1 (ng/mL)		
Control	4.65 ± 0.53	4.39 ± 0.73
Extract^b^	4.34 ± 0.49	3.23 ± 0.52

^a^Means significant statistical differences *p* < 0.05 between treatments

^b^Means significant statistical differences *p* < 0.001 intra-treatment

**Fig. 8:**
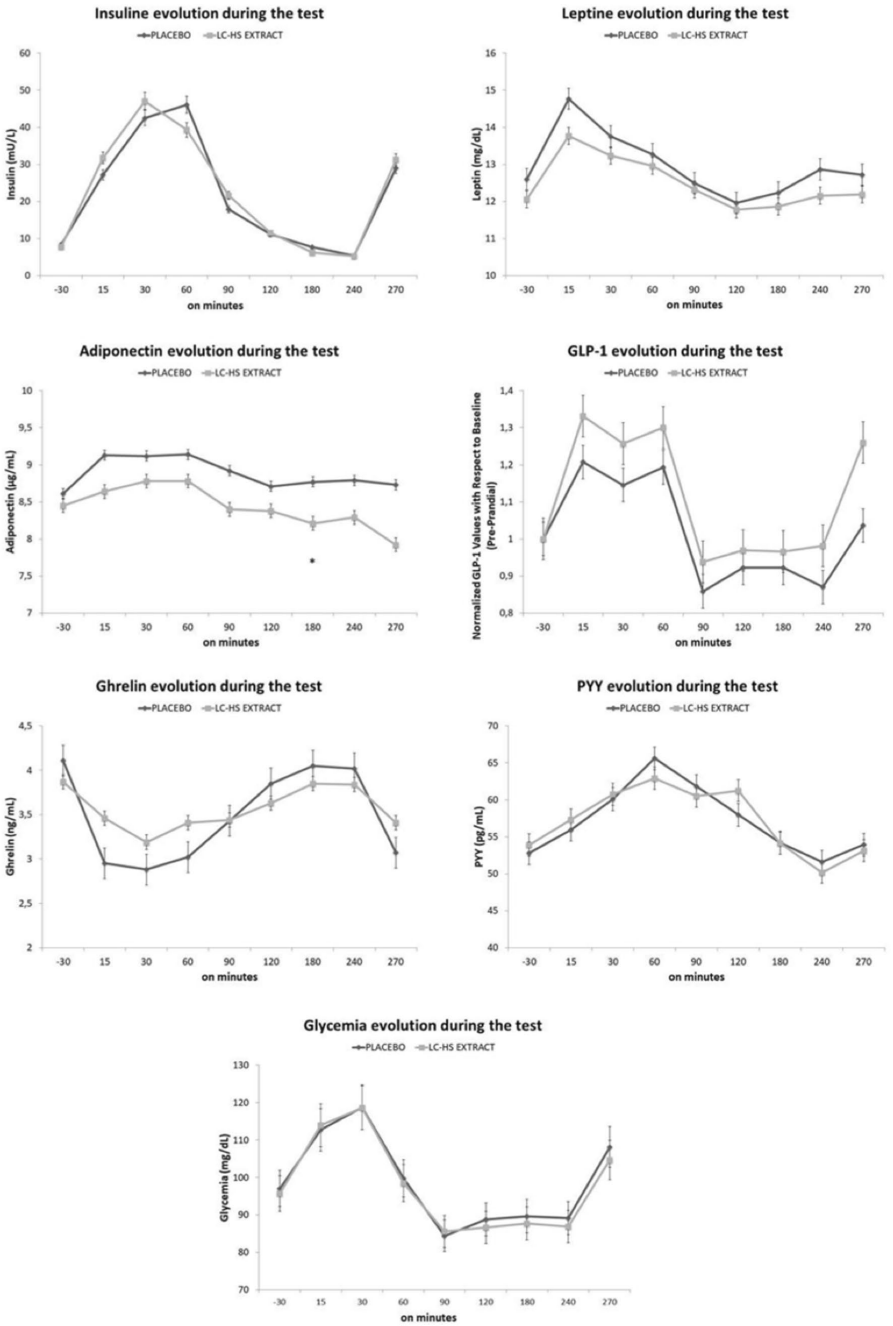
Hormones evolution during the study. *Means significant statistical differences between treatments *p* < 0.05

To the best of our knowledge, this is the first study to relate satiety measured by both hormones and satiety scales at different times during a stablished time period between meals. Considering evolution is important due to certain hormones such as ghrelin or GLP-1 have short active life span, regulating the amount of food consumed in a certain meal. Analyzing the evolution at different times during the test, there was not found significant difference between treatments in most of hormones (Fig. 8). However, plasmatic variation of adiponectin varied significantly at minute 180 during the test (*p* < 0.05), leading to higher concentration in subjects treated with Pla (8.77 ± 0.55 µg/mL) than Lc-Hs treatment (8.21 ± 0.5 µg/mL). GLP-1 values were normalized from baseline. Figure 8 shows the treatment with Lc-Hs as determinant factor for the increase of GLP-1 from min 15 (Δ 12.39%) Lc-Hs (1.33 ± 0.21) Pla (1.20 ± 0.19) until the end of the test (Δ 22.36%) Lc-Hs (1.26 ± 0.17) Pla (1.03 ± 0.16).

### Blood parameters

Obese and overweight population is commonly diagnosed with metabolic syndrome, so clinical and biochemical parameters need to be closely monitored. Table 3 shows glycemic and lipidemic profile of subjects at baseline and at the end of the study (long-term) from both treatments. At baseline, no significant differences were observed between treatments (*p* > 0.05) and remained stable after 8 weeks, so no significant changes were observed for the glycemic values during the study. Regarding HBA1c, the only remarkable variation occurred intra-treatment in the Lc-Hs treatment only (*p* < 0.009).

**Table 3:** Evolution of biochemical blood parameters of subjects during the study

	Baseline	Final
Glycemia (mg/dL)		
Control	95.13 ± 3.76	97.13 ± 3.31
Extract	94.12 ± 2.39	95.64 ± 2.07
HBA1c (%)		
Control	4.94 ± 0.07	5.01 ± 0.09
Extract^b^	4.98 ± 0.07	4.90 ± 0.06
HOMA-IR		
Control	1.92 ± 0.18	2.06 ± 0.19
Extract	1.75 ± 0.11	1.84 ± 0.13
Total cholesterol (mg/dL)		
Control	186.96 ± 5.91	187.47 ± 7.89
Extract	190.97 ± 5.55	182.90 ± 6.06
LDL (mg/dL)^a^		
Control	109.72 ± 5.17	113.08 ± 5.85
Extract	115.33 ± 5.85	109.98 ± 5.77
HDL (mg/dL)^a^		
Control	54.46 ± 1.37	53.46 ± 1.45
Extract	54.94 ± 1.42	57.46 ± 1.58
Triglycerides (mg/dL)		
Control	109.22 ± 6.23	109.06 ± 5.35
Extract	103.90 ± 5.94	104.25 ± 4.98

^a^Means significant statistical differences *p* < 0.05 between treatments

^b^Means significant statistical differences *p* < 0.009 *intra-treatment*

In turn, lipid profile showed minor but remarkable changes. There is a downward trend for cholesterol and LDL values after the consumption of the product under study. The plasmatic variation of LDL cholesterol was significantly minor in subjects from Lc-Hs treatment compared to Pla (*p* < 0.032). On the other hand, HDL cholesterol increased after the treatment with Lc-Hs compared to Pla, showing statistically significant differences (*p* < 0.008). However, triglyceride and total cholesterol values, remained at similar values than observed at baseline, regardless the treatment.

### Biompedance and body composition

**Fig. 9:**
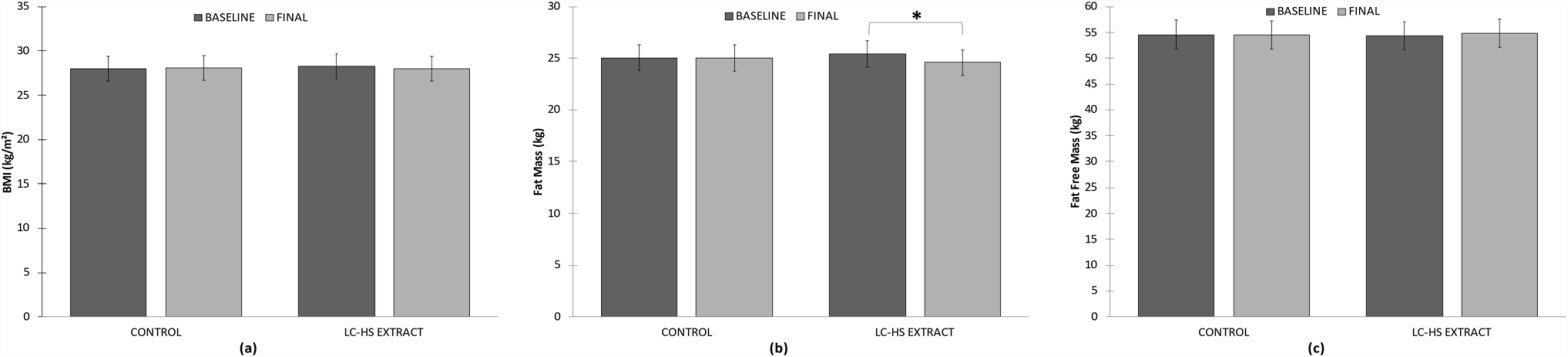
**a** Body mass index (kg/m^2^) evolution during study; **b** Fat mass (kg) evolution during study; **c** Fat free mass (kg) evolution during study. *Means significant statistical differences *p* < 0.002

Biompedance is a fast, secure, non-invasive and easy to apply method to evaluate body composition [43]. Biompedance values began in similar conditions for both treatments at baseline. BMI evolution did not show significant differences during the study, going from 28.03 ± 2.53 kg/m^2^ to 28.12 ± 2.56 kg/m^2^ in Pla and from 28.26 ± 2.52 kg/m^2^ to 27.98 ± 2.62 km/m^2^ showing a downward trend in Lc-Hs (Fig. 9).

According to the scientific literature, it is convenient to evaluate body weight in its different components, especially separate fat mass and fat-free mass (FFM) [44]. Figure 9 depicts evolution of fat mass and FFM, respectively.

Fat mass values evolution during the study were from 25.06 ± 7.68 kg to 25.04 ± 7.68 kg for Pla, and from 25.44 ± 7.41 kg for Lc-Hs Pla and 24.51 ± 7.14 for Lc-Hs) showing significant differences only in Lc-Hs *p* < 0.002 intra-subject. For FFM values evolution during the study were from 54.61 ± 10.82 kg to 54.53 ± 10.72 kg for Pla and from 54.44 ± 10.88 kg to 54.86 ± 11.08 kg for Lc-Hs not showing significant differences.

The results showed a reduction in fat mass in Lc-Hs treatment and maintenance of FFM and BMI compared to Pla.

### Physical activity

In order to reduce the error caused by personal differences in physical activity, sedentary subjects were exclusively recruited. The measurement of METs min/day by the accelerometer revealed that subjects from both placebo and experimental treatments maintained the same physical activity during the study. Regarding Pla treatment, values ranged (*p* = 0.418) from 1.7 ± 0.3 MET/min/day at baseline to 1.8 ± 0.3 MET/min/day at the end of the study. In turn, the experimental treatment showed similar values (*p* = 0.842) both at baseline (1.7 ± 0.4 MET/min/day) and at the end of the study (1.7 ± 0.3 MET/min/day). That fact supports that changes observed in subjects consuming the Lc-Hs extract may be a consequence of such consumption and not a change in physical activity habits.

## Discussion

The most noticeable effect observed during the monitoring of food intake was decreased satiety. Satiety is a necessary parameter regulating body weight control, since it is the most effective factor to achieve progressive and successful long-term weight loss. One of the most common factors during weight loss in obese or overweight patients is anxiety caused by a reduction in the volume of dietary food, which can lead to excessive intake. This situation is one of the main factors that compromise the effectiveness of the protocols of weight loss and lifestyle change established for the treatment of obesity and overweight.

The significant increase in the SQ (Δ 2.17%mm/kcal) observed in the present study positively correlates with the decrease in appetite sensation (Δ 11.76%) observed for the Lc-Hs group. That fact could explain the reason for the lower caloric intake of Lc-Hs group compared Pla group. As described in a recent systematic review [41], the SQ has clinically useful in relation to satiety response, and could be used as preventive measurement for the treatment and prevention of obesity. However, due to the heterogeneity of the methodology and the population context, it is difficult to make comparisons with other studies. Therefore, we believe that the unification of the different criteria used for the determination of SQ could lead to a better comprehension. In some studies, it was observed that SQ was predictive of food intake [39, 40, 45, 46], as can be observed in our study. In addition, the study of McNeil’s et al. [46] showed a positive correlation between SQ and changes in fat mass, as is reported in the present study.

Judging by the results of the present study, chronic intake of an Lc-Hs extract during 8 weeks increases satiety in overweight and obese population. The most noticeable effect was an increase in SQ, reducing daily caloric intake. It is important to note that higher values represent greater satiety and lower values show less satiety [47]. SQ is considered as solid indicator of satiety due to considers the AS before meals and the caloric content of food. In addition, has been shown to be positively associated with energy intake [38, 40] being the higher SQ value, the lower caloric consumption, as occurs in the present study. Another interesting insight is the observed decrease are AS during at least 2 h after meal intake, and reduced AUC from baseline to 240—being more pronounced from baseline to minute 60. Furthermore, due to similar protein content after *ad-libitum* intake in both treatments, it can be can affirmed that lower caloric intake and improvement in SQ and satiety do not relies on higher protein intake, which is known for its greater satiating capacity [11]. Recognizing that satiety is defined as the interval between meals as a function of elapsed time, and that it can be used to predict the next feeding episode [48], SQ in seems to predict ad-libitum intake in the next 3 h, a normal time period between meals.

Results observed for satiety and hunger sensation are in agreement with previous studies in overweight population that reported an improvement in satiety sensation the treatment with the same Hs-LC extract during 8 weeks [49]. Other authors have reported that different polyphenols can exert a synergistic effect to enhance their potential benefit [50, 51]. However, the bioavailability in the intestinal tract should be considered [52].

Research on polyphenols against obesity seems to be due to various mechanisms of action as; lower food intake, decrease lipogenesis, increase lipolysis, stimulate fatty acid *β*-oxidation, inhibit adipocyte differentiation and growth, attenuate inflammatory responses and suppress oxidative stress [53, 54]. For example, scientific evidence has sown that certain plant-derived such a *Hibiscus sabdarrifa or Lippia citriodora* can modulate different metabolic pathways and have certain effects activating the AMPK pathway, favoring lipolysis and fat loss [9, 17].

After analyzing the different blood samples, no long-term inter-subject significant differences were observed in different hormones such as insulin, adiponectin, PYY or Ghrelin. However, there are some tendencies that should be mentioned.

Ghrelin is synthesized in the gastric fundus and bloating of the stomach in the presence of a large volume of food [55] reduces its plasmatic concentration. In the present research, Ghrelin showed a downward trend that varies between Pla and Lc-Hs treatments. In fact, Pla treatment led to acutest diminution of ghrelin concentration during the 90 first minutes after meal intake. In turn, Lc-Hs treatment was not as effective for reducing ghrelin synthesis in the first 90 min after meal intake as Pla. However, it was able to achieve a homogeneous and stable decrease (plateau shape) along the 240 min of plasmatic monitorization. As observed previously, subjects treated with Lc-Hs reduced their caloric consumption, which can be a consequence of the reduction in the volume of food consumed that would lead to minor reduction on plasmatic ghrelin (greater plasmatic concentration). Therefore, Lc-Hs treatment was able to reduce caloric intake and food volume, while reducing Ghrelin concentration homogeneously for 240 min which can explain the reduction on hunger observed above. Despite not obtaining statistically significant results, it seems evident that treatment with Lc-Hs is able to reduce food intake, as shown in ad-libitum intake, appetite sensation and Ghrelin concentration in overweight and obese patients. The present data reinforce the idea that an adjuvant treatment with polyphenols can favorably affect glucose intake and regulation, adipogenesis, lipolysis, lipid metabolism, appetite control and improve pathologies related to obesity due to AMPK pathway modulation [9, 49]. As well as the practice of physical exercise that help to reach caloric deficit, may be the best strategy for the treatment of obesity given the nature of the disease [56].

The determination of Leptin resulted in decreased concentration in Lc-Hs treatment (*p* > 0.05), showing certain intra-subject significant decrease (*p* < 0.05) between baseline and final measurements. The same tendency was not observed in the acute part of the study due to the long-term effect of that hormone. Leptin is mainly synthesized in adipose tissue and is able to inhibit food intake, being the most important hormone for long-term maintenance of body weight. However, obese population may present leptin resistance, mainly due to lipid-related inflammation [57]. Considering this premise, the decrease in leptin levels can be explained by the loss of fat mass observed in patients treated with Lc-Hs, so that the secretory capacity of leptin would also be reduced due to a lower amount of adipose tissue. That reduction in adipose tissue could also be responsible of the decrease in low-grade inflammation that predominates in overweight or obese patient, which is part-responsible of leptin resistance [58]. Despite the reduction in leptin levels, the satiety sensation of the subjects treated with the Lc-Hs extract increased in comparison with the Pla treatment. This could be a consequence of reduced leptin resistance, which leads at least the same effects on hunger control with minor leptin secretion [9, 57].

The long-term study of adiponectin in the present study reported similar values than the previous literature regardless body fact variation along the long-term study, showing that caloric diet variation does not alter adiponectin levels over time [59, 60]. It has also been reported that low caloric diets do not varies plasmatic adiponectin levels regarding short [61, 62] and intermediate [63, 64] time periods. However, other authors reported that long-term interventions increase plasmatic adiponectin, improving abdominal fat distribution and lipid metabolism independently of weight change [65]. Short-term part of the present study reported continued reduction until the ad-libitum lunch for both Pla and Lc-Hs. Meanwhile, previous studies have seen an increase in adiponectin [66]. Therefore, the variation of adiponectin in the present study seems to be influenced in both short and long periods, showing different trends. However, the mechanism underlying adiponectin and its role in obesity needs more study to be considered.

Furthermore, in obese subjects, GLP-1 (anorexigenic satiating incretin) concentration is low, so a decrease could be expected in obese and overweight population [67, 68]. The acute part of the present study revealed similar values for both Pla and Lc-Hs treatments, with no differences between them. That fact will be different when the variation of chronic intervention is considered, as it will be commented below. Almost all the hormones presented similar baseline values, as shown in Fig. 8 to reach more significant results GLP-1 values was standardized from baseline. The standardization was necessary due to the differences observed at baseline that could be misleading. As can be observed, GPL-1 increased in the Lc-Hs treatment after the treatment, improving fullness and satiety that could explain the excellent SQ observed in that treatment. Therefore, in the present study, GLP-1 seems to be mayor factor controlling satiety and desire to eat.

In turn, the long-term part of the study showed some minor differences, but there were no noticeable. GLP-1 is synthesized in intestinal L cells whose secretion depends on the presence of nutrients in the lumen of the small intestine. Once GLP-1 reaches circulation, it has a half-life of a few minutes, due to rapid degradation by the enzyme dipeptidyl peptidase-4 [69]. Therefore, the long-term evolution of GLP-1 does not provide as much information about the control of hunger and satiety as does the short-term evolution. In fact, the scientific literature on the long-term effects of GLP-1 is an area yet to be explored.

Due to the improvements in lipid profile observed in in vitro an animal models [70], it seems desirable to measure these parameter in a human-based study. Meanwhile, glucose level was not improved during the present study; however, a significant reduction of glycated hemoglobin was observed in subjects treated with Lc-Hs but not in Pla treatment. In contrast, LDL Cholesterol and HDL Cholesterol varied significantly after the treatment with Lc-Hs, leading to minor and higher plasmatic levels, respectively. Meanwhile, total cholesterol showed a downward trend, but there was not found any significant variation for triglycerides. That variation in lipid profile may be due to the substances and antioxidant effects of the polyphenols present in Lc-Hs extract specially due to anthocyanins content [71, 72]. In this study, significant reductions in lipid levels were seen in hyperlipidemic subjects after consumption of *Hibiscus sabdariffa* [73].

Body composition was improved in the Lc-Hs treatment with a reduction in fat mass, not observing changes in FFM or BMI. It is essential to differentiate FFM from lean mass, due to many people misinterpret FFM value as muscle mass, overestimating that value [43]. FFM includes muscle mass, bone mass, skin mass, and residual mass. There were also no changes in quality of life test, as well as adverse events in liver or kidney function after product intake [74]. In this study, researchers found a reduction in fat mass (especially in the torso) after the consumption of 84 days of an Lc-Hs extract in similar population [75]. In the present study, the BMI of the subjects did not change along the whole study (*p* > 0.05).

Therefore, a polyphenolic extract based on *Lippia citriodora* and* Hibiscus sabdarrifa* may improve the regulation of appetite, mainly satiety, in overweight population. Moreover, the reduction in plasmatic leptin was expected due to reduction of the fat mass of subjects. In addition, the maintenance of FFM can produce a decrease in basal metabolism of the subjects, favoring a possible long-term weight reduction. This, together with hormonal and lipidemic changes observed in the study, may contribute to improvements in health.

The limitations of the study may be related with the metabolic variability of the participants since the range of BMI is relatively wide, so inflammatory status and gastric flexibility may differ between subjects. Moreover, the protocol determined for the measurement of satiety and related parameters—unless standardized and validated- is still relatively novel. Additional limitations corresponded to the low number of participants and the short time for intervention. Nevertheless, significant differences were observed when the two groups were compared at the end of the study.

## Conclusion

In conclusion, the consumption of 500 mg/day of mixed *Lippia citriodora* and* Hibiscus sabdarrifa* polyphenolic extract for 60 days in overweight subjects confirmed significant decrease on appetite sensation and body composition, with marked reduction of calorie intake during an ad-libitum meal, also improving lipidemic profile. Moreover, the increased satiety observed in the present study could be attributed to the changes observed in leptine, ghelin and GLP-1. However, despite the variation reported on hunger-related hormones, the present study was focused on satiety sensation but not on hormone regulation. Therefore, it would be interesting to develop future research with more appropriate methodology and focused on the variation of the hormones themselves as main research parameter.
